# Failure to rescue following anatomical lung resection. Analysis of a prospective nationwide database

**DOI:** 10.3389/fsurg.2023.1077046

**Published:** 2023-02-21

**Authors:** María Teresa Gómez-Hernández, Cristina Rivas, Nuria Novoa, Marcelo F. Jiménez, the Spanish Group of Video-assisted Thoracic Surgery (GEVATS)

**Affiliations:** ^1^ Service of Thoracic Surgery, Salamanca University Hospital, Salamanca, Spain; ^2^ Salamanca Institute of Biomedical Research, Salamanca, Spain; ^3^ University of Salamanca, Salamanca, Spain

**Keywords:** failure to rescue (FTR), lung resection, risk factor, postoperative complication, mortality, hospital volume

## Abstract

**Background:**

Rescue failure has been described as an important factor that conditions postoperative mortality after surgical interventions. The objective of this study is to determine the incidence and main determinants of failure to rescue after anatomical lung resections.

**Methods:**

Prospective multicenter study that included all patients undergoing anatomical pulmonary resection between December 2016 and March 2018 and registered in the Spanish nationwide database GEVATS. Postoperative complications were classified as minor (grades I and II) and major (grades IIIa to V) according to the Clavien-Dindo standardized classification. Patients that died after a major complication were considered rescue failure. A stepwise logistic regression model was created to identify predictors of failure to rescue.

**Results:**

3,533 patients were analyzed. In total, 361 cases (10.2%) had major complications, of which 59 (16.3%) could not be rescued. The variables associated with rescue failure were: ppoDLCO% (OR, 0.98; 95% CI, 0.96–1; *p* = 0.067), cardiac comorbidity (OR, 2.1; 95% CI, 1.1–4; *p* = 0.024), extended resection (OR, 2.26; 95% CI, 0.94–5.41; *p* = 0.067), pneumonectomy (OR, 2.53; 95 CI, 1.07–6.03; *p* = 0.036) and hospital volume <120 cases per year (OR, 2.53; CI 95%, 1.26–5.07; *p* = 0.009). The area under the curve of the ROC curve was 0.72 (95% CI: 0.64–0.79).

**Conclusion:**

A significant percentage of patients who presented major complications after anatomical lung resection did not survive to discharge. Pneumonectomy and annual surgical volume are the risk factors most closely related to rescue failure. Complex thoracic surgical pathology should be concentrated in high-volume centers to obtain the best results in potentially high-risk patients.

## Introduction

The term “failure to rescue” (FTR) was first described in 1992 by Silber et al. (1) and refers to the death of a patient after presenting a major postoperative complication. This metric has been proposed as an alternative parameter to postoperative morbidity and mortality rates to assess quality of care and to evaluate the performance of a surgical service or a hospital (2). For this reason, some institutions have already introduced FTR as a complementary indicator of surgical quality.

According to Silber et al. (1), while patientś features determine the occurrence of postoperative complications, hospital characteristics are associated to FTR. Thus, several studies (3–5) have related high rates of FTR to hospital factors such as a low volume of surgeries or high nurse-to-patient ratios. Additionally, according to Farjah et al. (6) the variation in mortality rates after pulmonary resection for lung cancer among hospitals is more related to the ability to rescue complicated patients than to the occurrence of complications.

On the other hand, although FTR is closely related to the ability to detect and treat complications early based on hospital features, some studies suggest that there are some patientś intrinsic factors that may increase the risk of FTR after complex surgeries with high rates of complications (7–11). Our hypothesis is that FTR is derived from a combination of patientś intrinsic factors and hospital features such as hospital volume.

Since FTR has been poorly investigated in thoracic surgery (2, 6) and no studies have been based on data obtained from a prospective nationally representative registry, we aimed to determine the incidence of FTR after anatomical lung resections and to investigate risk factors associated with FTR in a prospective nationwide multicenter setting.

## Methods

### Ethical statement

The Spanish Group of Video-Thoracoscopic Surgery (GEVATS) project (12) was approved by the ethics committees of all the participating centres and informed consent was obtained from the recruited patients to use their clinical data for scientific purposes (Approval by Ethics Committee of Aragon Health Research Institute on 20 May 2015 PI15/0072). This specific study was evaluated and approved by the scientific committee of GEVATS.

### Study design and data source

A prospective observational study was conducted based on the recorded data of the multicentre national registry of GEVATS. The registry contains information on all anatomical lung resections from December 2016 to March 2018. Database included data on patientś characteristics, surgical variables, and annual hospital volume of each participating center. Overall, 33 thoracic surgery departments participated in gathering data. The 283 variables extracted were stored in the MySQL database and adapted according to the Society of Thoracic Surgeons and the European Society of Thoracic Surgeons standardised definitions and terminology (13).

The data for this article were provided by GEVATS with permission. Data will be shared on request to the corresponding author with the permission of GEVATS.

### Primary outcome

The primary endpoint was failure to rescue, defined as any mortality occurring among patients who experienced a major postoperative complication within 30 days after the operation, or later if the patient was still in hospital.

Any adverse event occurring during admission or within 30 days after surgery was considered a postoperative complication. These complications were classified according to the Clavien-Dindo standardized classification of postoperative morbidity (14) into major (grade III: complications that require endoscopic or radiological surgical reintervention with or without general anesthesia; grade IV: complications that threaten the life of the patient and require treatment in intensive or intermediate care and grade V: complications that lead to the death of the patient) and minor (grade I: any deviation from the normal postoperative period that does not require reoperation, including the administration of electrolyte solutions, antiemetics, antipyretics, analgesics and physiotherapy and grade II: complications that require pharmacological treatment different from the above, including blood products and parenteral nutrition).

Patients who experience minor or no complications were excluded.

### Statistical analysis

Incidence of FTR was calculated by dividing the number of deaths among patients who experienced a major postoperative complication within 30 days after the operation, or later if the patient was still in hospital.

Potential risk factors for FTR were first analysed in a univariate logistic regression. Variables with a *p*-value < 0.2 were fed into a logistic regression model *via* stepwise backward elimination based on the Wald statistics. Absence of collinearity was checked by testing collinearity statics (tolerance and VIF). Finally, the discrimination of model was measured graphically with a ROC curve, and the goodness of fit of the model was assessed with the Hosmer-Lemeshow test.

Statistical analyses were performed using the statistical software SPSS 26 (IBM Corp, Chicago, Illinois, 2019).

Our manuscript is reported according to the STROBE and TRIPOD recommendations.

## Results

A total of 3,533 patients were recruited. Globally, 2,435 patients who had no complications and 737 patients who experienced minor complications were excluded from the analysis. The final sample consisted of 361 patients (10.2%) who had any major complication. Of these, 59 could not be rescued. Therefore, incidence of FTR was 16.3%.

Univariate analysis of patient characteristics, surgical features, and hospital volume for the dependent variable of FTR following pulmonary anatomical lung resections are shown in Table 1.

**Table 1:** Univariate analysis of patient characteristics, surgical features, and hospital volume for the dependent variable of FTR following pulmonary anatomical lung resections.

	Survivor (*n* = 302)	FTR (*n* = 59)	OR (CI 95%)	*p*-value
Age, mean (SD), years	66.02 (SD: 9.58)	68.63 (SD: 7.85)	1.034 (1.001–1.067)	0.045
Male sex, *n* (%)	239 (79.1)	53 (89.8)	2.33 (0.96–5.66)	0.062
BMI, mean (SD)	26.52 (SD: 4.55)	26.63 (SD: 4.28)	1.005 (0.994–1.071)	0.867
FEV1/FVC, mean (SD)	0.87 (SD: 0.2)	0.89 (SD: 0.13)	1.276 (0.293–5.557)	0.746
ppoFEV1%, mean (SD)	63.53 (SD: 16.85)	58.66 (SD: 17.05)	0.981 (0.964–0.998)	0.029
ppoDLCO%, mean (SD)	60.08 (SD: 17.81)	53.49 (SD: 16.23)	0.977 (0.959–0.995)	0.014
Tumoral size, mean (SD)	33.11 (SD: 21.86)	37.28 (SD: 25.74)	1.008 (0.995–1.020)	0.219
Smoking history, *n* (%)				0.405
– Never smoker – Former smoker – Current smoker – Unknown	27 (8.9)	2 (3.4)	1	
	175 (57.9)	40 (67.8)	3.09 (0.71–13.51)	
	96 (31.8)	16 (27.1)	2.25 (0.49–10.4)	
	4 (1.3)	1 (1.7)	3.36 (0.25–46.36)	
Cardiac comorbidity, *n* (%)	145 (48)	34 (57.6)	1.47 (0.84–2.59)	0.178
– Coronary disease – Arrythmia – Hypertension	32 (10.6)	10 (16.9)		
	27 (8.9)	5 (8.5)		
	123 (40.7)	33 (55.9)		
CKD, *n* (%)	8 (2.6)	3 (5.2)	2.01 (0.52–7.79)	0.315
Stroke, *n* (%)	14 (4.6)	4 (6.8)	1.5 (0.48–4.72)	0.492
Diabetes, *n* (%)	48 (15.9)	18 (30.5)	2.32 (1.23–4.38)	0.009
Peripheral arteriopathy, *n* (%)	34 (11.3)	9 (15.3)	1.42 (0.64–3.14)	0.388
Previous malignancy, *n* (%)	106 (35.1)	23 (39)	1.18 (0.67–2.1)	0.569
Diagnosis, *n* (%)			0.91 (0.5–1.67)	0.771
– Lung carcinoma – Pulmonary metastasis – Other	272 (90.1)	53 (89.8)		
	13 (4.3)	4 (6.8)		
	17 (5.6)	2 (3.4)		
Induction treatment, *n* (%)	25 (9.2)	5 (9.4)	1.03 (0.38–2.82)	0.995
Surgical approach, *n* (%)				0.011
– VATS – Open	126 (41.7)	14 (23.7)	1	
	176 (58.3)	45 (76.3)	2.3 (1.21–4.37)	
Pneumonectomy, *n* (%)	25 (8.3)	14 (23.7)	3.45 (1.67–7.13)	0.001
Extended resection, (%)	27 (8.9)	11 (118.6)	2.33 (1.09–5.02)	0.03
Lymphadenectomy, *n* (%)	270 (99.3)	52 (98.1)	0.39 (0.03–4.33)	0.439
pStage, *n* (%)				0.87
– I – II – III – IV	128 (48.9)	23 (50)	1	
	72 (27.5)	12 (26.1)	0.93 (0.44–1.97)	
	56 (21.4)	9 (19.6)	0.89 (0.39–2.06)	
	6 (2.3)	2 (4.3)	1.86 (0.35–9.76)	
Hospital volume (cases per year)*, *n* (%)				0.012
– ≥ 120 – < 120	125 (58.6)	14 (23.7)	1	
	177 (41.4)	45 (76.3)	2.27 (1.2–4.31)	

BMI: body mass index; FEV1: forced expiratory volume in the first second; FVC: forced vital capacity; ppoFEV1%: predicted postoperative forced expiratory volume in the first second; ppoDLCO%: predicted postoperative diffusing capacity of the lung for carbon monoxide; VATS: video-assisted thoracic surgery; SD: standard deviation; OR: odds ratio; CI: confidence interval.

* Hospital volume: 120 cases per year are equivalent to 150 cases during the study period (15 months).

Table 2 shows the results of the stepwise multivariable logistic regression analysis (dependent variable: rescue failure). In the final model, variables associated with FTR were: ppoDLCO% (OR, 0.98; 95% CI, 0.96–1; *p* = 0.067), cardiac comorbidity (OR, 2.1; 95% CI, 1.1–4; *p* = 0.024), extended resection (OR, 2.26; 95% CI, 0.94–5.41; *p* = 0.067), pneumonectomy (OR, 2.53; 95 CI, 1.07–6.03; *p* = 0.036) and hospital volume < 120 cases per year (OR, 2.53; CI 95%, 1.26–5.07; *p* = 0.009).

**Table 2:** Multivariate analysis of risk factors of FTR after anatomical lung resection.

	Adjusted OR (CI 95%)	*p*-value
ppoDLCO%	0.98 (0.96–1)	0.067
Cardiac comorbidity	2.1 (1.1–4)	0.024
Extended resection	2.26 (0.94–5.41)	0.067
Pneumonectomy	2.53 (1.07–6.03)	0.036
Hospital volume (cases per year), *n* (%)		0.009
– ≥120 – <120	1	
	2.53 (1.26–4.07)	

OR: odds ratio; CI: confidence interval; ppoDLCO%: predicted postoperative diffusing capacity of the lung for carbon monoxide; VATS: video-assisted thoracic surgery.

FTR rates among patients with cardiac disease history reached 19% and extended resection was associated with a 28.9% FTR rate. While 35.9% of patients who experienced any major postoperative complications following pneumonectomy died. Moreover, FTR rates in hospitals with low volume (<120 cases per year) was 20.3% against 10.1% in high-volume hospitals (≥120 cases annually).

**Figure 1:**
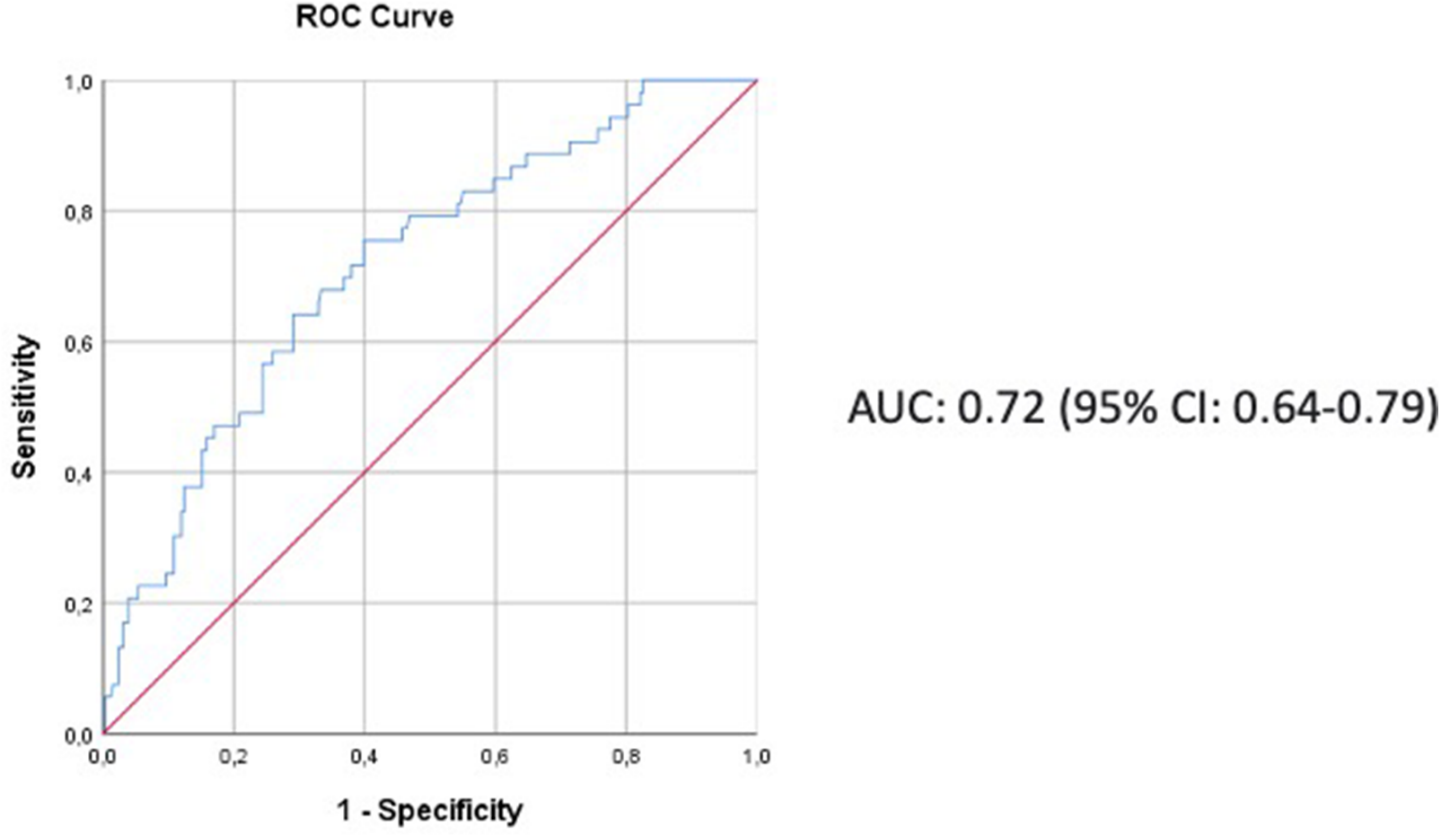
The ROC curve showing the discriminant power of the model.

The model showed acceptable levels of sensitivity and specificity: the area under the curve (95% CI) was 0.72 (0.64–0.79) (Figure 1) with a good degree of calibration (Hosmer-Lemeshow test value (*p* = 0.739).

## Discussion

The analyses of the nationwide prospective Spanish registry found that the incidence of mortality among patients who experienced any major postoperative complication following anatomical lung resection reaches 16.3%. This study shows similar failure to rescue rates after lung surgery to those previously reported in single institutional series (15).

In the current study, we identified several patient factors that combined with hospital volume may predict failure to rescue after anatomical lung resection. Prior research examining rescue failure rates in thoracic surgery focused more on characteristics of the institution rather than patient features (6, 16–19). In this regard, we found that FTR rates were 2-fold higher in low-volume hospitals in comparison to high-volume centres. Farjah et al. (6) also reported an important variation in FTR rates across hospitals. In their multicentre study based on the Society of Thoracic Surgeons General Thoracic Surgery Database, the risk of dying after major complications increased by 3-fold (20% vs. 6.9%, *p* < 0.001) across hospitals. Similarly, Tran et al. (19) reported that high-volume centre status was associated with reduced odds of mortality after re-intervention compared to low volume centres. Furthermore, Sanaiha et al. (20) found that high hospital lobectomy volume and minimally invasive approach decreased the odds of mortality after cardiovascular complications (myocardial infarction, cardiac arrest or pulmonary embolism). Additionally, minimally invasively approached patients at high-volume institutions had the lowest odds of all-cause mortality (OR 0.27) and myocardial infarction (OR 0.57). Several factors may contribute to such vastly different outcomes. According to Ghaferi et al. (21) teaching status, bed size and increased nurse-to-patient ratios may influence outcomes in patients undergoing pancreatectomy. Meanwhile, Ward et al. (22) suggested that improved intensive care services, rapid response teams and availability of personnel may have an important role in FTR outcomes. The combination of these factors at high-volume centres, may allow for an earlier detection and treatment of complications, thus rescuing patients from mortality; while, low-volume hospitals may lack these capabilities, which ultimately may explain the observed differences in outcomes.

However, although the hospital components are important to understand how to improve on these shortcomings, our purpose was to determine whether any inherent patient factors in combination with hospital volume were associated with mortality after postoperative complications. By identifying the high-risk patients, our efforts could then be focused on how to potentially prevent the occurrence of complications, how to rescue these patients after an adverse event or eventually referring high-risk patients to high-volume centres.

Our data demonstrate that ppoDLCO%, cardiac comorbidity, extended resection, and pneumonectomy in combination to hospital volume are the most important factors associated to FTR. These findings are consistent with prior studies illustrating the association of these factors with postoperative mortality after pulmonary resection. We previously reported in our institution (23) a 30-day mortality rate after pneumonectomy of 8.4% that reached 18.5% at six months after intervention. Among determinant factors of these results, we found age, laterality of the procedure and the occurrence of postoperative cardiorespiratory complications. Additionally, Dartevelle et al. (24) reported a mortality rate of 4% in T4 lung cancer patients undergoing extended resection after analysing a series of 388 cases operated in the last 30 years. Therefore, patients who may need a pneumonectomy or an extended resection should be thoughtfully selected, since some centres experience a high perioperative mortality rate, or referred to high-volume centres to guarantee optimal postoperative outcomes.

Several limitations need to be considered in this study. First, calculation of FTR was based on in-hospital deaths among patients who experience a major complication within 30 days after the operation, or later if the patient was still in the hospital. However, since a significant number of patients die after discharge within 90 days after pulmonary resection as a consequence of any surgical related complication (25), it can be considered that FTR based on 90-day mortality could be a better indicator of postoperative surgical care. Second, our data are based on a multicentre voluntary registry, so that, although the details of the data audit have been previously reported (12), bias related to patient selection and quality of data could have influenced our findings. Furthermore, our results should be confirmed in additional patient cohorts from different multi-institutional databases.

## Conclusion

16.3% of patients who presented major complications after anatomical lung resection in the GEVATS series did not survive to discharge. ppoDLCO%, cardiac comorbidity, pneumonectomy, extended resection, and annual surgical volume were the risk factors most closely related to FTR. Based on these results, complex thoracic surgical pathology should be concentrated in high-volume centres in order to obtain the best results in this type of potentially high-risk procedures.

## Data Availability

The raw data supporting the conclusions of this article will be made available by the authors, without undue reservation.

## The Spanish Group of Video-assisted Thoracic Surgery (GEVATS)

**Coordinator:** Raul Embún (University Hospital Miguel Servet).

**Scientific committee:** David Gómez de Antonio (University Hospital Puerta de Hierro Majadahonda, Madrid); Sergi Call (Mutua Terrasa University Hospital, University of Barcelona, Terrasa, Barcelona); Nicolás Moreno-Mata (Ramón y Cajal University Hospital, Madrid); Marcelo F. Jiménez (Salamanca University Hospital, University of Salamanca, IBSAL, Salamanca); Miguel Congregado (Virgen Macarena University Hospital, Seville); and Sergio Bolufer-Nadal (General University Hospital of Alicante).

**Local researchers:** José Luis Recuero and Íñigo Royo Crespo (University Hospital Miguel Servet and Lozano Blesa); Borja Aguinagalde and Iker López Sanz (Donostia University Hospital, San Sebastián-Donostia); Sergio Amor-Alonso and Francisco Javier Moradiellos-Díez (Quironsalud Madrid University Hospital, Madrid); Miguel Jesús Arrarás (Valencian Oncology Institute Foundation, Valencia); Ana Isabel Blanco Orozco (University Hospital Virgen del Rocío, Sevilla); Marc Boada and David Sánchez (Hospital Clinic of Barcelona, Respiratory Institute, University of Barcelona, Barcelona); Alberto Cabañero Sánchez (Ramón y Cajal University Hospital, Madrid); Isabel Cal Vázquez and Ramón Moreno-Balsalobre (University Hospital La Princesa, Madrid); Ángel Cilleruelo Ramos (University Clinical Hospital, Valladolid); Silvana Crowley Carrasco (University Hospital Puerta de Hierro Majadahonda, Madrid); Elena Fernández-Martín and Florentino Hernando Trancho (Hospital Clínico San Carlos, Madrid); Santiago García-Barajas and Cipriano López García (University Hospital of Badajoz, Badajoz); María Dolores García-Jiménez (University Hospital of Albacete, Albacete); José María García-Prim and Eduardo Rivo (Santiago de Compostela University Hospital, Santiago de Compostela); José Alberto García-Salcedo (12 de Octubre University Hospital, Madrid); Juan José Gelbenzu-Zazpe and María Elena Ramírez-Gil (Complejo Hospitalario of Navarre, Pamplona); Carlos Fernando Giraldo-Ospina and Roberto Mongil Poce (Hospital Regional Universitario, Málaga); María Teresa Gómez Hernández (Salamanca University Hospital, University of Salamanca, IBSAL, Salamanca); Jorge Hernández and Juan José Fibla Alfara (Sagrat Cor University Hospital, Barcelona); Jennifer D. Illana Wolf (Puerta del Mar Hospital, Cádiz); Alberto Jauregui Abularach (Vall d'Hebron University Hospital, Barcelona); Unai Jiménez and Rafael Rojo-Marcos (University Hospital Cruces, Bilbao); Néstor J. Martínez-Hernández (La Ribera University Hospital, Alcira, Valencia); Elisabeth Martínez-Téllez and Juan Carlos Trujillo Reyes (Santa Creu and Sant Pau Hospital, Autonomous University of Barcelona, Barcelona); Lucía Milla Collado (Hospital Arnau de Vilanova, Lleida); Sergio B. Moreno Merino (Virgen Macarena University Hospital, Seville); Carme Obiols (Mutua Terrasa University Hospital, University of Barcelona, Terrasa, Barcelona); Florencio Quero-Valenzuela (Virgen de las Nieves Hospital, Granada); Ricard Ramos-Izquierdo (University Hospital of Bellvitge, Hospitalet de Llobregat, Barcelona); Alberto Rodríguez-Fuster (Hospital del Mar, Hospital del Mar Medical Research Institute, Barcelona); Laura Sánchez Moreno (University Hospital Marqués de Valdecilla, Santander); Carlos Simón (Gregorio Marañón University Hospital, Madrid); and Julio Sesma Romero (General University Hospital of Alicante).
